# XIV International Entomophagous Insects Workshop: June 11–15, 2006, Newark, Delaware

**DOI:** 10.1673/031.007.1601

**Published:** 2007-03-29

**Authors:** Keith R. Hopper, Karen M. Kester, Kim A. Hoelmer

**Affiliations:** ^1^ USDA-ARS, Beneficial Insect Introduction Research Unit, Newark, Delaware, USA., khopper@udel.edu; ^2^ Department of Biology, Virginia Commonwealth University, Richmond, VA, USA., kmkester@vcu.edu; ^3^ USDA-ARS, Beneficial Insect Introduction Research Unit, Newark, Delaware, USA. khoelmer@udel.edu

Abstracts are listed in alphabetical order by the last name of the senior author.

