# Supplementary material for: XIV International Entomophagous Insects Workshop: June 11–15, 2006, Newark, Delaware
Source: J Insect Sci. 2007 Mar 29;7:16. doi: 10.1673/031.007.1601 (PMC2999420; doi:10.1673/031.007.1601)
Supplement: Related texts

# Research article: Parasitoids of *Myzus persicae* and *Aulacorthum solani* on pepper crops in British Columbia

**DOI:** 10.1673/031.007.1602

**Published:** 2007-03-29

**Authors:** Susanna Acheampong, David R. Gillespie, Don Quiring

**Affiliations:** Pacific agri-Food Research Centre (Agassiz); 6947 #7 Highway; P. O. Box 1000; Agassiz, BC V0M 1A0; Canada, acheampongs@agr.gc.ca

A survey of parasitoids of three clones of *Myzus persicae* (green peach aphid) and a clone of *Aulacorthum solani* (foxglove) on peppers in the lower Fraser valley of British Columbia, Canada from April — August 2005 revealed the presence of nine primary parasitoids. *Aphidius matricariae* was the dominant primary parasitoid species in all months, at all locations, and on all clones. Appearance of parasitoid species varied among parasitoid genera with some parasitoids occurring earlier in the season and others late in the season. Generally sex ratios were male-biased at the beginning of the season but as the season progressed, there was a shift towards a female-biased sex ratio. Hyperparasitoids were collected from three genera of hyperparasitoids, and were most common in the field in July and August.

# Research article: Using Magnetic Resonance Imaging (MRI) techniques to study parasitism in insect hosts

**DOI:** 10.1673/031.007.1603

**Published:** 2007-03-29

**Authors:** Marianne Alleyne, Luisa Ciobanu

**Affiliations:** ^1^ Department of Entomology, University of Illinois at Urbana-Champaign, Illinois, USA, vanlaarh@life.uiuc.edu; ^2^ Beckman Institute for Advanced Science and Technology, University of Illinois at Urban a-Champaign, Illinois, USA

The development of parasitoids within a host is usually visualized with the help of histological serial sections and subsequent reconstruction of the structures from these sect ions. A big drawback of this technique is the loss of the host and parasitoid, making studies of subsequent development of the same parasitized host impossible. Non-destructive imaging procedures could potentially give us new insights into how parasitoids survive and develop within a host. The goal of the present investigation was to assess the use of NMR imaging (MRI) for the study of endoparasitoid development. For this study we imaged *Manduca sexta* larvae parasitized by *Cotesia congregate* 6 to 12 days after parasitization and compared the images to those of unparasitized larvae. In order to limit manipulations larvae were not anaesthetized or injected with contrasting dyes. Larvae were contained within a tube for about 30 min during each imaging session. Experiments were performed using a 600 MHz NMR spectrometer. 600 MHz represents one of the highest frequencies at which animal micro-imaging experiments have been successfully performed to date. This frequency level affords the maximal spatial resolution and contrast currently available. We were able to obtain images from larvae on consecutive days. Parasitoids emerged from parasitized larvae successfully, even after 3 or more imaging sessions. Images clearly showed different regions, within the body as a whole and within the 1 mm imaging-slice, containing primarily water or fat molecules. However, we were unable to distinguish between parasitized and unparasitized larvae, presumably because the fat in a parasitoid larva is indistinguishable from fat within the fat body using this technique.

# Research article: Mortality and fertility of *Aphelinus near parmali* at four temperatures

**DOI:** 10.1673/031.007.1604

**Published:** 2007-03-29

**Authors:** Jay Bancroft

**Affiliations:** USDA-ARS, 17053 N. Shatter Ave., Shatter, CA 93263 USA, jsbancroft@pw.ars.usda.gov

This biocontrol agent has over-wintered at several locations in California's Central Valley, but population abundance has not taken off. ANP had a survival time of 15, 9, 5, 2 d at 21, 26, 31, 36 °C, respectively. Single day mummy production was greatest at 26 °C. Development time was 20, 17, 13 d at 21, 26, 31 °C, respectively. Somewhat lower parasitization occurred on cotton aphid than green peach aphid, on which the colony is maintained. Although ANP had poor reproductive success at 36 °C, further studies of fluctuating temperatures and resource use may elucidate how to provide refuges and increase population spread of this wasp in the hot, dry habitat.

# Research article: Plant and prey consumption cause a similar reduction in cannibalism by an omnivorous bug

**DOI:** 10.1673/031.007.1605

**Published:** 2007-03-29

**Authors:** Mika Beck, Moshe Coll

**Affiliations:** Department of Entomology, the Hebrew University of Jerusalem, Rehovot, 76100, Israel, mikabeck@gmail.com

Cannibalism is usually more intense when other food sources are scarce, be it prey or plant-based foods. We hypothesized that feeding on plants would reduce cannibalism to a lesser extent than feeding on prey, because plants are considered nutritionally inferior compared to prey. We used the omnivorous bug *Orius laevigatus* Say (Heteroptera: Anthocoridae) to test this prediction. Starved female bugs were individually held with five second-instar conspecific nymphs and offered (i) *Helicoverpa armigera* eggs (prey); (ii) pollen (plant); (iii) *H*. *armigera* eggs and pollen (prey + plant); or (iv) no eggs or pollen. Fewer cannibalistic events and shorter feedings on conspecifics were recorded in the presence of pollen, prey or both than in their absence. The Data therefore do not support our hypothesis that cannibalism is differentially affected by foods of different nutritional values. It seems that omnivorous feeding habits enable predators to sustain themselves on plant sources in the absence of prey, without the need to resort to cannibalism.

# Research article: Oviposition behavior of *Neodusmetia sangwani* (Hymenoptera: Encyrtidae) in an invasive host, *Antonina graminis* (Hemiptera: Pseudococcidae), and potential impacts on the red imported fire ant, *Solenopsis invicta* (Hymenoptera: Formicidae)

**DOI:** 10.1673/031.007.1606

**Published:** 2007-03-29

**Authors:** Jillian M. Chantos, Ken R. Helms, S. Bradleigh Vinson

**Affiliations:** Department of Entomology, Texas A—M University, College Station, TX, USA, jc32004@neo.tamu.edu

*Neodusmetia sangwani* was introduced from India in the 1950′s as a biological control agent of the invasive mealybug *Antonina graminis,* a common pest of Texas rangeland grasses. The red imported fire ant, *Solenopsis invicta*, tend *A*. *graminis* for their honeydew secretions resulting in a faster colony growth rate. Understanding the potential quadratrophic interaction involving Bermuda grass, *Cynodon dactylon*, *A*. *graminis*, *N*. *sangwani*, and the red imported fire ant, *S*. *invicta* could result in reduced colony numbers of *S*. *invicta* by limiting the amount of available carbohydrates. In this study oviposition behavior of *N*. *sangwani* is an important link in reducing colony numbers of *S*. *invicta* by controlling *A*. *graminis* populations. A general chemical analysis was conducted to identify behavior chemicals that *N*. *sangwani* use in host identification of *A*. *graminis.*

# Research article: Use of host-size-dependent sex ratio theory in parasitoid mass rearing

**DOI:** 10.1673/031.007.1607

**Published:** 2007-03-29

**Authors:** Andrew Chow, Kevin M. Heinz

**Affiliations:** Department of Entomology; Texas A—M University; 2475 TAMU; College Station, TX 77843-2475, USA, achow@tamu.edu

An inherent problem with augmentative biological control is the high cost for the large numbers of control agents needed for suppressing pests. Overproduction of males in parasitic Hymenoptera contributes to higher costs for biological control because only females kill pests directly. We present a technique, based on manipulation of female parasitoid behavior and host composition, for generating less male-biased sex ratios in parasitoid species that adjust their sex allocation in response to relative host size. Our technique used predictions generated from host-size-dependent sex ratio theory to develop a system for decreasing the proportion of males produced in mass rearing of *Diglyphus isaea* (Walker) (Hymenoptera: Eulophidae), a commercially available parasitoid of agromyzid leafminers. We present findings on: (1) mechanisms used by female ale wasps for assessment of host size, (2) how host-size information is used to make sex allocation decisions, (3) how sex allocation behavior can be manipulated in a commercially feasible manner, and (4) differences in wasp quality when female production is maximized. First, we identified behaviors and measurements made by female wasps that correlate well with the probability that a given sex will be laid. We then asked whether naive or experienced wasps assessed host size differently.

Two parameters were important for predicting sex allocation: wasp transits and time for host paralysis. Wasp experience affected the extent but not the types of parameters that predict sex allocation. Second, we used host quality and memory models to show how female wasps are influenced more by recent than past host encounters in their assessment of host size for sex allocation. Third, we compared the offspring sex ratios of female wasps presented with different compositions of hosts. Presenting females with increasingly larger hosts over 1–2 days reduced mean sex ratio; however, females produced more male-biased sex ratios (similar to commercial units) if exposed continuously to only large hosts. Increasing host size over time has limited application for insectaries. Thus, we tested whether sex allocation could be modified by the availability and distribution of two different-sized hosts. We then compared the use of both small hosts and large hosts to only large hosts for simulated mass rearing of wasps over 8 weeks. Using both small hosts and large hosts produced similar numbers of wasps as using only large hosts, but reduced mean sex ratio of weekly cohorts from 66% male to 56% male. The two techniques produced females of similar size, but using both small hosts and large hosts produced slightly smaller males than using only large hosts. Finally, we compared control of agromyzid leaf miners with releases of identical numbers and sex ratios of wasps produced by the two rearing technique. In trials simulating infestation of greenhouse chrysanthemums during an 11-week crop cycle, we found no significant differences between the levels of control obtained with wasps produced by either rearing technique. Adoption of our rearing technique by commercial insectaries could reduce implementation costs for not only *D*. *isaea* but also other parasitoids that show host-size-dependent sex allocation.

# Research article: Chemical analysis of residues left by walking adults of *Nezara viridula* which induce arrestment behavior in the egg parasitoid *Trissolcus basalis*

**DOI:** 10.1673/031.007.1608

**Published:** 2007-03-29

**Authors:** Stefano Colazza, Gloria Aquila, Ezio Peri, Jocelyn Miliar

**Affiliations:** ^1^ Department of S.En.Fi.Mi.Zo., Section of Acarology, Entomology and Zoology, Università di Palermo, Italy, colazza@unipa.it; ^2^ Department of Entomology, University of California — Riverside, USA

Chemical residues deposited by walking adults of the southern green stink bug (SGSB), *Nezara viridula* (L.) (Heteroptera: Pentatomidae), which play a role as contact kairomones inducing arrestment behavior in the egg parasitoid *Trissolcus basalis* (Wollaston), were investigated in laboratory experiments. Female wasps encountering an area contaminated by chemical residues from adult hosts showed an arrestment response characterized by prolonged periods of walking and turning, resulting in systematic return to the stimulus after encountering the treatment borders. When SGSB adults were dissected into separate body parts, extracts from legs and the dorsal laminar pronota of adult fermales elicited equal responses, whereas extracts of legs and dorsal laminar pronota of adult males elicited lower responses. These results corroborate previous results showing the wasp's preference for chemical residues from female hosts, and they suggest that contact kairomones are not confined to the legs, but are distributed over the adult cuticula. Hexane extracts of laminar pronotua and legs of SGSB adults induced stronger responses than methanol extracts. Silica gel chromatography of SGSB cuticular extracts indicated that the activity was associated with the fraction containing saturated and unsaturated hydrocarbons. The potential significance of these results to the host location behavior of *T*. *basalis* in the field is discussed.

# Research article: Omnivory and spatial dynamics reduce adverse effect of intraguild predation on herbivore suppression

**DOI:** 10.1673/031.007.1609

**Published:** 2007-03-29

**Authors:** Moshe Coll, Sulochana Shakya, Phyllis Weintraub

**Affiliations:** ^1^ Department of Entomology, The Hebrew University of Jerusalem, Rehovot, Israel, coll@agri.huji.ac.il; ^2^ Entomology Unit, Gilat Research Center, Negev, Israel

Most terrestrial food webs include intraguild (IG) predators, which feed on herbivores as well as other predators in the system. In general, IG predators have been shown to suppress intermediate predator populations, allowing herbivore densities to increase. Yet many of the interacting predators are in fact omnivores that feed on prey as well as plant-based foods, such as flower pollen. We therefore hypothesized that omnivorous feeding acts to reduce the adverse effect of intraguild predation (IGP) on herbivore suppression and lessen plant damage. That pollen becomes available on some plant structures (flowers) but not others, adds another important level of complexity to these systems. We tested these trophic interactions in a system that consisted of strawberry plants, western flower thrips (WFT, *Frankliniela occidentalis*) and two of its predators, the mite *Neoseiulus cucumeris* and the bug *Orius laevigatus*. All three consumers feed on strawberry pollen, WFT damages strawberry fruits, the mite preys on 1st instar WFT, and the bug feeds on WFT and the mites. Results show that (i) significantly fewer WFT were killed by the predators in the presence of pollen than in its absence; (ii) predation on *N*. cucumeris by *O*. *laevigatus* (i.e., IGP) was significantly lower in the presence of pollen than in its absence; (iii) in the presence of pollen, WFT and both predators primarily reside in flowers rather than fruits and leaves; (iv) In the absence of pollen, WFT were recorded primarily on fruits; (v) In the presence of pollen, *N*. cucumeris is found in the flowers only when *O*. *laevigatus* is absent; else, the mites are found on the fruits or leaves; and (vi) When both predators are present, significantly lower fruit damage was observed in the absence of pollen than in its presence. Taken together, results show that omnivorous feeding and differential response to spatially heterogeneous resources buffer strong herbivorous and IG predatory interactions. This may allow for complex trophic interactions, such as omnivory and IGP, to persist and be common in nature.

# Research article: Symbiofauna Associated to the Reproductive System of *Cotesia flavipes* and *Doryctobracon areolatus* (Hymenoptera, Braconidae)

**DOI:** 10.1673/031.007.1610

**Published:** 2007-03-29

**Authors:** F.L. Cônsoli, E.W. Kitajima

**Affiliations:** Department of Entomology, ESALQ, University of São Paulo, Piracicaba, São Paulo, 13418-900, Brasil, fconsoli@esalq.usp.br

The diversity of symbionts associated to insects and the multitude of effects on their host biology has prompt a number of studies to understand the role these microorganisms may have on host biology and how they affect their host interactions with other trophic levels. There is also a possibility of using such symbionts as vectors of genes for insect control or learning on the mechanisms they use to interact with their hosts for the development of new approaches to insect control. As most of them are transmitted transovarially from one generation to another, we investigated the presence and distribution and characterized the morphology of such microorganisms in the female reproductive tissues of two important insect pest parasitoids, the braconids *Cotesia flavipes* and *Doryctobracon areolatus*, through electron microscopy. *C*. *flavipes* was found to be associated with a polydnavirus (PDV), while *D*. *areolatus* harbored the rickettsia-like bacterium Wolbachia. We also provide a discussion on the role of these symbionts on the population biology of both parasitoids.

# Research article: Multiple locus complementary sex determination in the parasitoid wasp *Cotesia vestalis*

**DOI:** 10.1673/031.007.1611

**Published:** 2007-03-29

**Authors:** Jetske G. de Boer, Paul J. Ode, Louise E.M. Vet, Jim Whitfield, George E. Heimpel

**Affiliations:** ^1^ Department of Entomology, University of Minnesota, 219 Hodson Hall, 1980 Folwell Ave., St. Paul, MN 55108 USA, deboe039@umn.edu; ^2^ Department of Entomology, North Dakota State University, 270 Hultz Hall, 1300 Albrecht Blvd., Fargo, ND 58105 USA; ^3^ Netherlands Institute of Ecology (NIOO-KNAW), Villa Vijverhof, Rijksstraatweg 6, 3631 AC Nieuwersluis, The Netherlands and Laboratory of Entomology, Wageningen University, Binnenhaven 7, 6709 PD Wageningen, The Netherlands; ^4^ Department of Entomology, University of Illinois, 320 Morrill Hall, 505 S. Goodwin Ave., Urbana, IL 61801 USA

Among the Hymenoptera, single locus sex determination (sl-CSD) describes a mode of sex determination in which females develop from fertilized eggs that are heterozygous at a single polymorphic sex locus. Males are haploid when they develop from unfertilized eggs or diploid when they develop from fertilized eggs that are homozygousat the sex locus. These diploid males are commonly inviable or sterile. This genetic load associated with sl-CSD is especially severe under conditions of inbreeding or low diversity of sex alleles. Since the 1970′s it has been hypothesized that sl-CSD might be a specific case of multiple locus CSD (ml-CSD). Under ml-CSD, diploid males would develop only from fertilized eggs that are homozygous at each of two or more sex loci. Ml-CSD thus results in much lower fractions of diploid males than sl-CSD and can therefore significantly reduce the genetic load on a population. We have now found evidence for ml-CSD in the parasitoid wasp *Cotesia vestalis* (= *C*. *plutellae*). This conclusion is based on patterns of sex ratio and fractions of diploid males over multiple generation of inbreeding.

# Research article: Analysis of dynamic, replicated, quantitative food webs

**DOI:** 10.1673/031.007.1612

**Published:** 2007-03-29

**Authors:** Eric Dyreson, M.L. Henneman

**Affiliations:** ^1^ Dept. of Mathematics, University of Montana-Western, Dillon MT 59725 USA, e_dyreson@umwestern.edu; ^2^ Dept. of Environmental Sciences, University of Montana-Western, Dillon MT 59725 USA

Quantitative food web analysis is a useful tool for assessing ecosystem change over time due to species invasions, biocontrol projects, habitat loss and global warming. With the advent of rapid data collection technologies, the emerging field of data-based analysis of food web dynamics will play a crucial role in long-term prediction, management decisions, and resource allocation. We propose a comprehensive methodology for gathering and analyzing quantitative food web data over time which will allow researchers to test hypotheses concerning the magnitude of change over time. We use previously collected food-web data and model food webs replicated both in space and time to demonstrate the utility of multivariate analysis of dynamic, replicated, quantitative (DoRQ) food webs for the detection of changes in entire webs and subwebs, and of phenomena such as indirect effects, which are increasingly recognized as an important but overlooked potential problem with species introductions.

# Research article: Analysis of dietary proteins derived from prey eggs and embryonic cell lines and their effects on the fecundity of *Orius insidiosus* (Hemiptera: Anthocoridae)

**DOI:** 10.1673/031.007.1613

**Published:** 2007-03-29

**Authors:** Stephen M. Ferkovich, J.P. Shapiro, Dwight E. Lynn

**Affiliations:** ^1^ USDA-ARS, Center for Medical, Agricultural, and Veterinary Entomology, 1700 SW 23rd Dr., Gainesville, FL 32604 USA, sferkovich@gainesville.usda.ufl.edu; ^2^ Insect Biocontrol Laboratory, USDA/ARS, BARC-West, Bldg. 011A, Rm. 214, Beltsville, MD 20705-2350 USA

Laboratory-reared predators fed on artificial diets often demonstrate reduced fecundity relative to those fed on prey. The reason for reduced egg production is not clear. Knowledge of the basic mechanisms governing the reproductive processes (e.g. vitellogenesis, oogenesis, oviposition) of predators is needed to improve existing artificial diets and to better understand their reproductive strategies in the field. In contrast to those fed artificial diets, *O*. *insidiosus* and other heteropteran predators fed on prey contain more yolk protein and produce more eggs. In many cases, the low fecundity of females reared as nymphs on artificial diets can be reversed by feeding the resulting adults on live prey. Consequently, we have hypothesized that high quality prey eggs and cell lines contain a fecundity-enhancing factor. We have analyzed protein extracts from eggs of *Ephestia kuehniella* and *Plodia interpunctella*, and from two embryonic cell lines, EK-x4V and PiE, from those respective species. Fractions obtained from electrophoretic and chromatographic separations of the proteins were bioassayed in artificial test diets and compared against an optimal standard, *Ephestia* eggs, and control diet on the basis of oviposition rates. Active fractions isolated from both *Ephestia* and *Plodia* eggs and the PiE cell line produced significant improvements in oviposition rate over the control diet. Efforts are continuing toward identification of factors.

# Research article: Influence of host size upon the fitness of *Eretmocerus mundus*

**DOI:** 10.1673/031.007.1614

**Published:** 2007-03-29

**Authors:** Dan Gerling, Roni Shoshan, Moshe Guershon

**Affiliations:** Tel Aviv University, Department of Zoology; Ramat Aviv, Israel 69978, dangr@post.tau.ac.il

Insect size is usually considered dependent on nutritional resources, positively affecting fitness parameters such as eggload and longevity. In koinobiont parasitoids such resources may be dynamic and change during the period of parasitoid immature development. The parasitoid *Eretmocerus mundus* develops in hosts that may differ greatly in size. These size differences are usually unapparent during oviposition under the 2nd–3rd whitefly nymphs and materialize mainly during the 4th instar of the host. We examined the size ranges of the emerging parasitoids in relation to host size and their corresponding fitness expressed in longevity, survival, egg load and number of eggs laid. The results showed that smaller whitefly nymphs (as measured by their exuvial length) produced smaller whitefly adults (expressed as tibial length). However, parasitoid size was indifferent to an increase in host size, possibly excluding the extremes of exceptionally small or large individuals. Positive correlations were found between parasitoid tibial length and eggload, number of eggs and longevity. Measurements taken in a greenhouse serving for mass-rearing of *E*. *mundus* showed no differences in size distributions of parasitoid adults that had just emerged from their pupae and those collected as adults from the greenhouse. Thus, there appears to be no differential survival of larger parasitoid individuals within the greenhouse. Since no apparent relationship was found between host and parasitoid size, we conclude that the lack of discrimination of potential host sizes is not a primary factor affecting parasitoid size and fitness. However, other growth influencing parameters, such as the host stage at parasitisation or nutritional status (with consequences in parasitoid developmental time) may influence its size and fitness and therefore should be considered for mass-rearing efforts.

# Research article: Evolution of the complementary sex determiner gene: A phylogenetic perspective on complementary sex determination in the Hymenoptera

**DOI:** 10.1673/031.007.1615

**Published:** 2007-03-29

**Authors:** Karl Gruber, Jetske G. de Boer, George E. Heimpel

**Affiliations:** 1 Department of Ecology, Evolution and Behavior, University of Minnesota, St. Paul, MN 55108 USA, grub0121@umn.edu; ^2^ Department of Entomology, University of Minnesota, St. Paul, MN 55108 USA

Complementary sex determination (CSD) is the mechanism thought to regulate sex in haplodiploid Hymenopteran insects (bees, wasps, ants, and sawfly). Under CSD an egg becomes female only if heterozygous at a sex locus, otherwise it becomes a male, and it has been identified for a wide variety of species. The molecular basis of this mechanism was recently found to be controlled by a single gene named complementary sex determiner (csd), identified in the honeybee. However, for most species little or nothing is known about the molecular basis of their reproductive mode, regardless of whether they exhibit the CSD phenotype or not. We build upon the discovery of the csd gene and employ a phylogenetic approach to study the evolution of csd across the Hymenoptera. We designed primers based on published csd sequences of *Apis* *mellifera*, *A*. *cerana*, *A*. *dorsata* and *Melipona compressipes* and used them to amplify a fragment of the csd gene spanning exons 2 and 3 in representative taxa of the Hymenoptera. Our study gives new insight into the evolution of the csd gene across the phylogenetic landscape of the Hymenoptera. Our results also hold promise for the development of a fast and efficient PCR based approaches for the identification of the CSD phenotype in prospective biological control agents.

# Research article: The importance of vision in locating prey within a patch for a true omnivore

**DOI:** 10.1673/031.007.1616

**Published:** 2007-03-29

**Authors:** T.R.J. Hazard, D. Gillespie, B.D. Roitberg

**Affiliations:** ^1^ Department of Biological sciences, Simon Fraser University, Burnaby, BC, V5A 1S6 Canada, thazard@sfu.ca; ^2^ Pacific Agri-Food Research Centre, Agriculture and Agri-Food Canada, POB 1000 Agassiz, BC V0M 1A0 Canada

*Dicyphus hesperus* Knight (Heteroptera: Miridae) is a true omnivore that is used in tomato greenhouses to control soft-bodied arthropod pests. True omnivores, i.e., those that consume both plant and animal material, must locate both prey and plant food and it is not clear which sensory modalities are used in doing so. It is generally accepted that strict predators rely on visual cues to some degree to locate prey. Here we investigate the importance of visual cues for *D*. *hesperus*. This research should provide insights into ways of manipulating the environment to improve the efficacy of *D*. *hesperus*.

# Research article: Efficiency of endemic *Trichgramma* species of olive farms to control the olive (*Prays oleae*) and jasmine (*Palpita unionalis*) moths in Egypt

**DOI:** 10.1673/031.007.1617

**Published:** 2007-03-29

**Authors:** E.M. Hegazi, A. Herz, S. Hassan, W. Khafagi, E. Agamy, A. Zaitun, S.M. Abdel-Rahman, S. El-Said, N. Khamis

**Affiliations:** ^1^ Faculty of Agriculture, Alexandria University, Egypt, eshegazi@hotmail.com; ^2^ Institute for Biological Control, Darmstadt, Germany; ^3^ Plant Protection Research Institute, Sabahia, Alexandria, Egypt; ^4^ Faculty of Agriculture, Cairo University, Egypt; ^5^ Central Lab of Pesicides, Cairo, Egypt

Augmentative releases of locally occurring *Trichogramma* [*T*. *euproctidis* (TEU), *T*. *cordubensis* (TC) and *T*. *bourarachae* (TB)] were evaluated against the olive and jasmine moths. Parasitization by the endemic species was compared with both natural parasitization and commercially available [*T*. *evanescens* (TE)]. The study has shown that mass releases of endemic species significantly (P<0.01) increased egg-parasitism in comparison with parasitism in non-release or TE-releases. The following could be detected: 1) TB had a higher efficiency in parasitizing *P*. *oleae* eggs than other wasps, 2) releases of TC increased significantly the parasitization of *P*. *unionalis* eggs, 3) the variability of parasitism data between the endemic species (TC, TB and TEU) and TE was significant, 4) TC parasitize *P*. *unionalis* eggs more frequently than *P*. *oleae* eggs, 5) the results suggest that *P*. *unionalis* act as a reserve store for Trichogramma wasps to attack latter the P. oleae eggs and vice versa, 6) TB and TC were more potential than the other two species in parasitization of unequal numbers of *P*. *oleae* and *P*. *unionalis* eggs, 7) the percentage of infestation of the fruits that fell by *P*. *oleae* or *P*. *unionalis* was lower in sites treated by endemic species than in other sites, and 8) fruit harvest/tree was significantly greater in sites treated with endemic species than other sites.

# Research article: Are the earlier instars better defended immunologically than later instars? A case study of immune dynamics in the *Microplitis ryfiventris*-*Spodoptera littoralis* system

**DOI:** 10.1673/031.007.1618

**Published:** 2007-03-29

**Authors:** Esmat Hegazi, Wedad Khafagy

**Affiliations:** ^1^ Department of Economic Entomology, Faculty of Agriculture, Alexandria University, Egypt, eshegazi@hotmail.com; ^2^ Plant Protection Research Institute, Sabahia, Alexandria, Egypt

Single and superparasitization experiments were carried out using *Microplitis rufiventris* Kok. (Braconidae : Hymenoptera) to study the dynamics of stage-dependent immune responses in *Spodoptera littoralis* (Boisd.) larvae (Lepidoptera : Noctuidae). The later instars (non-preferred hosts) displayed vigorous “humoral response” and often damaged and destroyed the single wasp's egg or larvae compared to the earlier (preferred) instars. The variable range of superparasitization and host age at parasitism caused changes in both cellular and “humoral immune” responses. Earlier hosts showed stronger encapsulation response compared with later ones. Also, the encapsulation rate in earlier hosts (e.g., second instar) decreased drastically as the number of deposited eggs/larva increased. In later instars, the encapsulation response was low in fourth, weak in fifth and no response in sixth instar hosts. The order and magnitude of cellular immune response in *S*. *littoralis* hosts were second instar most, first, third and fourth next and fifth and sixth instar least. While those of “humoral immune” response were sixth instar larvae most, fifth and fourth next and first, third and second ones least. The results suggest that both cellular and humoral responses are stage-dependent. The outcome of immune response of *S*. *littoralis* larvae toward the surplus wasp larvae resulted in wasps of siblicidal solitary development in earlier host instars and a nonsibicidal behaviour in the penultimate (fifth) and (sixth) host instars. It is speculated that differences in juvenile hormone and its homologues in sixth versus earlier instar hosts may account for the apparent reduction of physical/physiological suppression among competing wasp larvae. If this is true, this would be a unique mechanism promoting the spread of gregariousness development in wasp species.

# Research article: Using ‘parasitization rates’ to determine natural enemy impact: What are we measuring?

**DOI:** 10.1673/031.007.1619

**Published:** 2007-03-29

**Authors:** M.L. Henneman, Jane Memmott

**Affiliations:** ^1^ Department of Environmental Sciences, University of Montana-Western, Dillon MT 59725 USA, l_henneman@umwestern.edu; ^2^ School of Biological Sciences, Woodland Road, University of Bristol, Bristol, U.K. BS8 1UG

The term ‘parasitization rate’ has not been clearly defined, despite being commonly used in the literature. In a meta-analysis, we found that some studies measured exclusively attack rates of parasitoids on hosts, while the majority measured exclusively emergence rates. Only occasionally were both of these measured. Although they are related, neither attack nor emergence alone can be used to define the interaction between parasitoid and host. Emergence rate is equal to attack rate minus parasitoid death rate in the host (killing the host as well), minus death rate in the host without host death (usually via encapsulation). From the host's perspective, total mortality is equal to attack rate minus encapsulation rate. Total mortality is also equal to emergence rate plus death rate (killing the host). In the context of testing the slow-growth, high-mortality hypothesis, we use our own rearing and dissection data to show that using emergence as a proxy for ‘parasitization rate’ to estimate the impact of parasitoids on a host, while ignoring parasitoid attack, can be misleading.

# Research article: Flexible behaviour and co-adapted gene complexes in a generalist parasitoid

**DOI:** 10.1673/031.007.1620

**Published:** 2007-03-29

**Authors:** Lee M. Henry, Bernard D. Roitberg, David R. Gillespie

**Affiliations:** ^1^ Department of Biological sciences, Simon Fraser University, Burnaby, BC, V5A 1S6 Canada Agassiz, BC V0M 1A0, lmhenry@sfu.ca; ^2^ Pacific Agri-Food Research Centre, Agriculture and Agri-Food Canada, POB 1000

Plasticity in adult body size allows generalist parasitoids to utilize many host species at a cost of producing a range of adult sizes. Consequently, host selection behaviour must also maintain a level of flexibility as adult size is related to capture efficiency. In the present study we investigated co-variance of two plastic traits — size at pupation and host size selection behaviour — using *Aphidius ervi* reared on either *Acyrthosiphon pisum* or *Aulacorthum solani*, generating females of disparate sizes. Our objectives were to determine if natal host influenced the oviposition success rate and host size preference of females when choosing between instars of each host species, given that there is an approximate two-fold difference in the optimal sized host instar between the two aphid species. Results are discussed in the context of co-adapted plastic traits that are closely associated with productive fitness and the evolution of phenotypic linkage in variable environments.

# Research article: Ecology of the olive fruit fly and its parasitoids in wild olives in Southern Africa

**DOI:** 10.1673/031.007.1621

**Published:** 2007-03-29

**Authors:** K.A. Hoelmer, A.A. Kirk, C.H. Pickett

**Affiliations:** ^1^ USDA-ARS Beneficial Insects Introduction Research Unit, Newark, Delaware, USA, khoelmer@udel.edu; ^2^ European Biological Control Laboratory, USDA-ARS, Montferrier-sur-Lez, France; ^3^ California Department of Food & Agriculture, Biological Control Program, Sacramento, California, USA

The olive fruit fly, *Bactrocera oleae* (Rossi) (Diptera; Tephritidae) is a key pest of cultivated olives throughout the Mediterranean region, where it has no effective natural enemies. Olive flies are specific to *Olea europaea*, of which various subspecies occur widely in parts of Africa, southern Europe and Asia as far east as China. Field surveys conducted in the 20th century documented a diverse assemblage of natural enemies on cultivated and wild olives in southern and eastern Africa, but previous attempts to introduce these species into Europe were not successful. The recent establishment of *B*. *oleae* in California has renewed interest in classical biological control of this pest. To identify and obtain new natural enemies of olive fly for evaluation and potential establishment in California, we surveyed wild olives, *Olea europaea* subsp. *cuspidata*, in several regions of South Africa (RSA) and Namibia during 2001–2005. Wild olives have a wide distribution across southern Africa, but rainfall patterns strongly influence the seasonal occurrence and abundance of fruit, and consequently the abundance of flies and their parasitoids. The annual pattern of rainfall in South Africa varies among regions, leading to a wide range in flowering and fruiting phenology. Olives tend to set fruit more frequently and produce more numerous and larger fruits in the moister regions of West Cape Province, RSA, than in drier regions. Olive fly populations were consistently highest in West Cape Province, as were populations of their natural enemies. Drier regions support populations of olive fly at much lower levels. In drier habitats the fruit is less likely to ripen. Fruit size and pulp thickness also influences fly infestation rates. Wild olive fruits possess relatively thin pulp (ca. 1–3 mm), offering little food for fly larvae. Pulp thickness is relevant for biological control, because cultivated olives have much thicker pulp in which fly larvae may be able to escape the reach of parasitoids equipped with relatively short ovipositors. Several braconid species in the genera *Bracon*, *Psyttalia* and *Utetes* were the most abundant fly parasitoids reared from infested olive fruit collected in southern Africa. The dominant species varied from region to region and from one year to the next. Braconid parasitoids were often present even at low densities of olive fly. The parasitoid fauna of southern Africa was similar to that found in Kenya but much richer than the diversity known from North Africa.

# Research article: Evolution of reproductive incompatibility and courtship behavior in cryptic species of aphid parasitoids

**DOI:** 10.1673/031.007.1622

**Published:** 2007-03-29

**Authors:** Keith R. Hopper, Angela M. I. De Farias, Seth C. Britch, James B. Woolley, John M. Heraty, Roberto Romani

**Affiliations:** ^1^ UDA-ARS, Newark, Delaware, USA, khopper@udel.edu; ^2^ Federal University of Pernambuco, Recife, Brazil; ^3^ Department of Entomology, Texas A&M University, College Station, Texas, USA; ^4^ Department of Entomology, University of California, Riverside, California, USA; ^5^ Department of Agricultural and Environmental Sciences, University of Perugia, Italy

Mapping insect behavior on a phylogeny can reveal how behavior has evolved. DNA sequences provide useful traits for determining phylogenetic relationships, especially among species that differ little in morphology. Phylogenetic patterns in mating behavior among closely related species may reveal how species originated. As part of projects on biological control of aphids, we studied populations of parasitic wasps in the *Aphelinus varipes* complex from various geographical regions and host species. A molecular phylogeny showed that each population was also a separate lineage. In laboratory experiments, females from each source were exposed to males from the same and other sources and left together with appropriate species of aphids for their lifetimes. The proportion of couples with female offspring differed greatly among crosses: most populations did not produce female offspring when crossed. Although distant lineages were mostly reproductively isolated and close lineages compatible, there were exceptions: the species from *D*. *noxia* in Georgia was isolated from its sister species in France and Israel, but it was not isolated by behavior from the Asian lineages. To test for differences in courtship behavior, we exposed females to males from each source and observed pursuit, courtship, acceptance, copulation, and mate guarding. Males from each source courted females from every other source, but females from most sources rejected copulation and insemination by males from other sources. There were no differences in sequence or durations of pursuit, courtship, copulation, or mate guarding among sources. However, male antennation behavior did differ among sources. Male antennae have glands whose secretions may affect female recognition of appropriate mates.

# Research article: Evolution and genetics of host use in cryptic species of the *Aphelinus varipes* complex

**DOI:** 10.1673/031.007.1623

**Published:** 2007-03-29

**Authors:** Keith R. Hopper, Seth C. Britch, James B. Woolley, John M. Heraty

**Affiliations:** ^1^ USDA-ARS, Newark, Delaware, khopper@udel.edu; ^2^ Department of Entomology, Texas A&M University, College Station, Texas, USA; ^3^ Department of Entomology, University of California, Riverside, California, USA

Populations of parasitoids from different host species or geographical regions often differ in host specificity. In some cases, these populations have been shown to represent cryptic species. Here, we present results on the evolution and genetics of host specificity in the *Aphelinus varipes* (Hymenoptera: Aphelinidae) species complex. A molecular phylogeny and crossing experiments showed that populations of *A*. *varipes* from the aphid hosts *Diuraphis* *noxia*, *Ropalosiphum padi*, and *Aphis glycines* (Homoptera: Aphididae) in France, Georgia, Israel, China, Korea, and Japan included five cryptic species. These species differed in parasitism of seven aphid species in five genera and two tribes on four host plant species in no-choice laboratory experiments. Some species showed narrow to monospecific host use, others attacked most or all host species tested. Most species were reproductively isolated by pre-zygotic, behavioral barriers involving female choice. However, some allopatric species were partially or completely reproductively compatible in laboratory crosses, although they differed in host specificity, morphology, and DNA sequences. Using this compatibility, we introgressed genes for use of a novel aphid species from one parasitoid species to another in laboratory crosses, and we used these crosses to map quantitative trait loci (QTL) involved in host specificity. Two QTL explained 7 percent of the variation in parasitism of the novel host.

# Research article: Hyperparasitoids increase patch time in an Aphid Parasitoid

**DOI:** 10.1673/031.007.1624

**Published:** 2007-03-29

**Authors:** Evelyne Hougardy, Nick J. Mills

**Affiliations:** Department of Environmental Science, Policy and Management Division of Organisms & Environment, Mulford Hall, University of California Berkeley, CA 94720 USA, ehougard@nature.berkely.edu

The foraging behavior of primary parasitoids can be influenced by the presence of hyperparasitoids, sometimes reducing patch time allocation and resource exploitation. We examined whether the walnut aphid parasitoid, *Trioxys pallidus* (Haliday) shows a similar response. Two generalist hyperparasitoid species were tested.

# Research article: Imperfect Preference for Singly parasitised hosts over doubly parasitised hosts in the semi-solitary parasitoid *Echthrodelphax fairchildii*: Implications for profitable self-superparasitism

**DOI:** 10.1673/031.007.1625

**Published:** 2007-03-29

**Authors:** Emi Ito, Yoshihiro Y. Yamada

**Affiliations:** Insect Ecology Laboratory, Faculty of Bioresources, Mie University, Tsu, Mie 514-8507, Japan, yamada-y@bio.mie-u.ac.jp

When conspecific female adults prefer singly parasitised hosts over doubly parasitised hosts, self-superparasitism can be profitable (positive in fitness returns) or more profitable. We examined whether such preference occurs in the small brown planthopper *Laodelphax striatellus* (Fallén) (Homoptera: Delphacidae) and its semi-solitary infanticidal parasitoid *Echthrodelphax fairchildii* Perkins (Hymenoptera: Dryinidae). Furthermore, the adaptiveness of the preference was assessed by comparing the value (fitness gain from oviposition) of doubly parasitised hosts with that of singly parasitised hosts. The first-to-second and second-to-third intervals in triple parasitism were 1 or 24 h, and the interval in double parasitism was the first-to-third interval in the corresponding triple parasitism. Triple and double parasitism occurred with similar frequencies irrespective of the oviposition intervals although the value of doubly parasitised hosts was lower than that of singly parasitised hosts for all four oviposition intervals. However, when doubly parasitised hosts were categorized into two types; ones with the first and second comers on the same side and ones with the two comers on different sides, female parasitoids preferred the former over the latter for all oviposition intervals, and they also preferred singly parasitised hosts over the latter except triple parasitism with oviposition interval s of 24/1 h. There was no preference between doubly parasitised hosts with the first and second comer on the same side and singly parasitised hosts. The value of singly parasitised hosts and that of doubly parasitised hosts with the two comers on the same side were equally higher than that of doubly parasitised hosts with the two comers on different sides, irrespective of the oviposition intervals. This indicates that females did not always exhibit a preference between singly and doubly parasitised hosts with different values. The main cause for no preference was considered to be lack of discrimination ability.

# Research article: Identification of parasitoid species using PCR amplification and restriction enzyme digestion

**DOI:** 10.1673/031.007.1626

**Published:** 2007-03-29

**Authors:** Violaine Jourdie, Nadir Alvarezand, Ted Turlings

**Affiliations:** Université de Neuchâtel, Institut de Zoologie, Laboratoire d'Entomologie Evolutive, Emile-Argand 11, CP 158, 2009 Neuchâtel, Switzerland, violaine.jourdie@unine.ch

*Spodoptera frugiperda* is a pest of great economic importance in the Americas. It is attacked by several species of parasitoids, which act as biological control agents. Parasitoids are morphologically identifiable as adults, but not as larvae. Laboratory rearing conditions are not always optimal to rear out parasitic wasps from *S*. *frugiperda* larvae collected from wild populations, and it frequently happens that parasitoids do not complete their life cycle and stop developing at the larval stage. Therefore, we explored ways to identify parasitoid larvae using molecular techniques. Sequencing is one possible technique, yet it is expensive. Here we present an alternate, cheaper way of identifying seven species of parasitoids (*Cotesia marginiventris*, *Campoletis sonorensis*, *Pristomerus spinator*, *Chelonus insularis*, *Chelonus cautus*, *Eiphosoma vitticolle* and *Meteorus laphygmae*) using PCR amplification of COI gene followed by a digestion with a combination of four restriction endonucleases. Each species was found to exhibit a specific pattern when the amplification product was run on an agarose gel. Identifying larvae revealed that conclusions on species composition of a population of parasitic wasps can be biased if only the emerging adults are taken into account.

# Research article: Courtship songs of the *Cotesia flavipes* complex

**DOI:** 10.1673/031.007.1627

**Published:** 2007-03-29

**Authors:** Andrea L. Joyce, R. E. Hunt, S. B. Vinson, J. S. Bernal

**Affiliations:** ^1^ Dept. of Entomology, Mail Stop 2475, Texas A&M University, College Station, Texas 77843 USA; ^2^ Dept. of Biology, Indiana University Southeast, 4201 GrantLine Road, New Albany, Indiana, 47150 USA, ajoyce@neo.tamu.edu

*Cotesia* is a genus of parasitoid wasps (Hymenoptera: Braconidae) that has been widely used for biological control. The *Cotesia flavipes* complex contains three species, *C*. *flavipes*, *C*. *sesamiae* and *C*. *chilonis*, which are difficult to distinguish morphologically. *Cotesia flavipes* is distributed throughout the Indo-Australian region, *C*. *sesamiae* is found in Africa, and *C*. *chilonis* has been collected from Japan and China. These parasitoids have been used for biological control in over 40 countries for stem boring lepidopteran pests such as *Chilo* and *Diatraea* spp., which damage crops including rice, sugar cane, maize and sorghum. Our objective was to determine if courtship signals differed among the three members of this species complex. Courtship songs have been employed successfully to distinguish among morphologically similar insects in other orders such as Orthoptera, Neuroptera and Hemiptera. Male *Cotesia* species wing fan while courting females, and wing fanning produces a low amplitude sound and substrate vibration. Two recording methods were compared for recording these songs. A condenser microphone was used to record airborne sound, while laser vibrometry recorded substrate vibrations. The courtship songs of males of the *C*. *flavipes* complex were recorded, as was a more distantly related species, *Cotesia marginiventris*. Signal analysis software was used to determine the duration, frequency, and intervals between signal components from the recordings. The signals of the species recorded were found to be significantly different. Implications for biological control will be discussed.

# Research article: Local adaptation and beyond: What does learning have to do with it?

**DOI:** 10.1673/031.007.1628

**Published:** 2007-03-29

**Authors:** Karen Kester, Amanda Lentz, Bonnie Brown

**Affiliations:** Department of Biology, Virginia Commonwealth University, Richmond, VA 23284 USA, kmkester@vcu.edu

The behavior of insect parasitoids that utilize phytophagous hosts reflects past selective pressures exerted by plants. Optimally, parasitoids should locate and make the greatest reproductive investment in hosts feeding on plants most likely to favor the survival and fecundity of their offspring. For parasitoids attacking lepidopteran larvae encountered on more than one plant species, reproductive investment should be focused but not limited to hosts encountered on the parasitoid natal plant. To test this hypothesis, we quantified the effects of sequentially learned plant cues on searching responses and ovipositional decision-making in *Cotesia congregate*. (Say) (Hymenoptera: Braconidae), a gregarious larval endoparasitoid of the solanaceous specialist, *Manduca sexta* L., and other sphingids. Most sphingids are plant family specialists and our previous work has demonstrated population differentiation of *C*. *congregata* with respect to host/foodplant utilization. In the present study, parasitoids received a postemergence learning experience (= early adult learning) with one of two food plants (tobacco or tomato) of host *M*. *sexta* or with one of two novel plants (parsley or arugula), and a subsequent ovipositional experience with the previously experienced or alternate plant. Parasitoids had no other contact with plants. Hosts were of uniform stage and size, and reared exclusively on a semi-synthetic diet. Effects of the two learning experiences were additive and results for host and novel plants differed. Experience with host plants induced an increased searching response to the plant/plants experienced and a proportionately greater allocation of female progeny to hosts offered with the plant(s) experienced, but had no effect on clutch size allocation decisions. In contrast, experience with novel plants induced an increased searching response to the plant/plants experienced and mixed effects on clutch size, but had no effect on sex ratio allocation decisions. This study demonstrates the importance of both inherent and learned plant cues on ovipositional decision-making in insect parasitoids, and suggests a potential mechanism for population differentiation with respect to host/foodplant utilization.

# Research article: *In vitro* rearing of *Toxoneuron nigriceps* (Hymenoptera: Braconidae), a larval endoparasitoid of *Heliothis virescens* (Lepidoptera: Noctuidae) from early second instar to third instar larvae

**DOI:** 10.1673/031.007.1629

**Published:** 2007-03-29

**Authors:** Indira Kuriachan, Fernando D. Consoli, S. Bradleigh Vinson

**Affiliations:** ^1^ Department of Entomology, Texas A&M University, College Station, Texas 77843 USA, indira@tamu.edu; ^2^ DepartAmento de Entomologia, Fitopatologia e Zoologia Agricole, ESALQ — Universidade de Sao Paulo, Piracicaba, SP13418-900, Brazil

The early second instar larvae of *Toxoneuron nigriceps*, a larval endoparastioid of *Heliothis virescens*, were incubated in artificial rearing media, supplemented with hemolymph of the unparasitized and parasitized fifth instar larvae of the host, *Heliothis virescens*. The parasitoid larvae were incubated in both a semisolid and liquid form of the artificial rearing medium, and their growth and development were evaluated. The growth in size (increase in length and width), development (molting), and survival of the incubated larvae were observed for ten days. The incubated larvae exhibited some level of growth in all nine types of media tested, including the control (without host hemolymph). However, ingesting the semisolid rearing media supplemented with the hemolymph from the late fifth instar (day 5, 7 and 9) parasitized host resulted in 100% of the larvae molting to third instars. Some of the in vitro reared third instar larvae demonstrated behavioral changes that could be interpreted as the preparation for cocoon formation or pupation i.e. oral secretion of a whitish material and lots of twisting and turning; however, none produced a cocoon nor pupa.

# Research article: Battle of the sexes in the parasitoid *Copidosoma sosares*

**DOI:** 10.1673/031.007.1630

**Published:** 2007-03-29

**Authors:** Evan Lampert, Paul Ode

**Affiliations:** Department of Entomology, North Dakota State University, Fargo, ND 58105 USA

The haplodiploid genetic system of Hymenoptera results in relatedness asymmetries between males and female siblings; females share on average 25% of genes with brothers, and males share on average 50% of genes with sisters. Furthermore, polyembryonic species such as *Copidosoma sosares* produce genetically identical broods of 100–350 “siblings.” *C*. *sosares* produces single-sex (r=1) and mixed-sex broods. Competitive asymmetries between the sexes, biased against males, are expected in larvae of this species, which consume completely the lepidopteran host. Brood composition and tibia length were recorded from over 500 broods either collected from U.S. and European natural populations or reared on artificial diet. Male tibiae were 0.02mm longer in all-male broods than in mixed-sex broods, in which developing male larvae competed against female siblings. In contrast, lengths of female tibiae were not significantly different between all-female or mixed-sex broods. Also, tibia length of both sexes decreased as clutch size increased in mixed-sex broods, but not in single-sex broods. Finally, sex ratio (proportion male) in mixed-sex broods varied from 0.125–0.92 and increased with increasing clutch size. These data begin to suggest that developing larvae of different sexes compete asymmetrically for limited host resources.

# Research article: Influence of plant morphology on the efficacy and intraguild interactions of pea aphid predators

**DOI:** 10.1673/031.007.1631

**Published:** 2007-03-29

**Authors:** Ana Legrand

**Affiliations:** Department of Plant Science, U-4067, 1376 Storrs Road, University of Connecticut, Storrs, CT 06269 USA, ana.legrand@uconn.edu

Tri-trophic level interactions have been shown to be mediated by chemical and physical plant characteristics. It has been possible to test hypotheses regarding the effect of plant morphology variation on the effectiveness and intraguild interactions of pea aphid predators by using pea near-isogenic lines. These lines exhibit single gene mutations that drastically alter the shape of leaves and stipules while minimizing changes in other plant characteristics. Three near isogenic lines (Normal leaf, acacia leaf and parsley leaf) were used to test the hypothesis the predation efficacy of *Coccinella septempunctata* larvae and *Chrysoperla rufilabris* larvae will be reduced when the predators forage on plants with increased morphological complexity. Increased leaf morphological complexity was found to be detrimental to the predation efficacy of *C*. *septempunctata* larvae. However, the efficacy of *Chrysoperla rufilabris* larvae was unaffected by increases in complexity. This difference in predator responses was also observed when predators foraged on peas exhibiting leaf type (afila leaf and Normal) and stipule type (Normal and reduced) combinations. Predation by chrysopid larvae was significantly enhanced only when afila leaf occurred together with reduced stipules. Conversely, coccinellid larvae consumed more aphids while foraging on plants with afila leaves regardless of stipule type and while on plants with reduced stipules independent of leaf type. In addition to influencing the efficacy of predation on aphids, variation in plant morphology legislated the occurrence of intraguild predation among the aforementioned insect predators. Pairs of coccinellid and chrysopid larvae were placed on three plant types (afila leaf, Normal leaf and parsley leaf) that ranged in morphological complexity. It was hypothesized that coccinellid larvae will survive better when they are on the complex parsley leaf plants because of their reduced range of movement while on these plants. Coccinellid larvae experienced a significant increase in survival from chrysopid predation when they were on the parsley leaf plants compared to the other plant types.

# Research article: A framework to assess predator response to herbivore-induced plant volatiles

**DOI:** 10.1673/031.007.1632

**Published:** 2007-03-29

**Authors:** David C. Margolies, James R. Nechols, Punya Nachappa

**Affiliations:** Department of Entomology, Waters Hall, Kansas State University, Manhattan, KS 66506-4004 USA, dmargoli@ksu.edu

Recently there have been exciting advances in the study of herbivore-induced plant volatiles and their role in tritrophic interactions. Of particular interest for biological control are studies to clarify the impact of herbivore-induced plant volatiles on prey reduction in the field through the attraction of natural enemies. However, attraction to herbivore-induced plant volatiles occurs within a broader context of many foraging and oviposition choices; so we need to examine the response to volatiles in concert with other traits if we intend to successfully understand these complex interactions. We have been studying the interaction between the herbivorous mite, *Tetranychus urticae* Koch, and its predator, *Phytoseiulus persimilis* Athias-Henriot, in a tritrophic context. Our ultimate goal is to assess the impact of behavioral mechanisms on demographics in this system and to predict the population dynamics at an ecologically-relevant level. We have focused on four predator traits which are likely to be critical for predator fitness and predator-prey dynamics in patchy environments: consumption rate, conversion efficiency, patch leaving rate, and attraction to/arrestment by prey-induced plant volatiles. We have approached this study from several directions, which will be discussed in this presentation. First, we will present information on predator behavior within a patch in response to interacting influences from several environmental cues. Second, we will discuss the use of quantitative (phenotypic) genetic techniques to measure the genetic contribution to, and correlations among, the above mentioned traits. Third, we will describe experiments using lines of *P*. *persimilis* selected for high or low levels (relative to an un selected population) of these traits to directly examine their impact on predator-prey dynamics under various situations.

# Research article: Phenotypic and genetic differentiation of hymenopteran parasitoids

**DOI:** 10.1673/031.007.1633

**Published:** 2007-03-29

**Authors:** Raul F. Medina, Pedro Barbosa

**Affiliations:** ^1^ Department of Entomology, Texas A&M University, 2475 TAMU, College Station, TX 77843 USA, rfmedina@tamu.edu; ^2^ Department of Entomology, University of Maryland, College Park, MD 20742 USA

The ecology of parasitoids is strongly influenced by the host-plants of their hosts. Parasitoid fitness is affected by a variety of plant traits. Thus, it is reasonable to assume that adaptation to plant traits could promote phenotypic and genotypic differentiation among populations of parasitoids. We present data on phenotypic and genetic differences of two braconid parasitoids ovipositing on the same host species on different host-plant species. We compare adult mass, adult longevity, and percent parasitism of a generalist (*Cotesia marginiventris* Cresson, Hymenoptera: Braconidae) and of a specialist parasitoid (*Aleiodes nolophanae* Ashmead, Hymenoptera: Braconidae) of the green cloverworm (*Plathypena scabra* Fabricius, Lepidoptera: Noctuidae) on alfalfa and soybean and use AFLP markers to assess genetic differences in parasitoids emerging from the same host species on alfalfa and soybean plants.

# Research article: Single versus multiple introductions, and differential success in the biological control of homopteran and lepidopteran pests

**DOI:** 10.1673/031.007.1634

**Published:** 2007-03-29

**Authors:** Nick Mills

**Affiliations:** Department of Environmental Science, Policy and Management, Mulford Hall, University of California, Berkeley, CA 94720-3114 USA, nmills@nature.berkeley.edu

Two of the most challenging questions in biological control are whether the single best natural enemy will provide greater suppression of the abundance of an arthropod pest than a combination of natural enemies, and why homopteran pests have been far more successful target for biological control than lepidopteran pests. Parasitoids and predators that share a common host are frequently involved in interspecific interactions that appear to be indicative of intense competition. There is also clear evidence from the biological control record that competitive displacement of both parasitoids and predators is a real, though not particularly frequent, outcome of multiple introductions of natural enemies. Although there is some evidence that competition may limit the success of establishment of natural enemies, there is little evidence that it influences the success of pest suppression. In addition, numerous examples of coexistence among natural enemies in both natural and production ecosystems provide overwhelming evidence for the avoidance of interspecific competition, with both experimental and theoretical evidence suggesting that this most likely arises through environmental heterogeneity and the spatial partitioning of natural enemy niches. Biological control has few unifying principles and no theoretical basis for understanding the differential pattern of success against homopteran and lepidopteran pests. Despite the fact that there are numerous differences in the ecological and life history characteristics of these two groups of pests and their associated parasitoids, few have the potential to explain their differential susceptibility to success in biological control. From a theoretical perspective, we have identified two life history traits, generation time ratio and gregarious development, that could substantially improve the impact of parasitoids in biological control. There is also evidence from the biological control record that both generation time ratio (Homoptera) and gregarious parasitoid development (Lepidoptera) are more frequently represented among biological control successes than failures. Thus despite the potential for tradeoffs between these and other fitness traits in the life history evolution of parasitoids, generation time ratio coupled with a broad window of host attack would appear to be the most likely mechanism to account for differential success in the biological control of homopteran and lepidopteran pests.

# Research article: Effects of vegetation management in field margins on *Meteorus autographae* and *Pseudoplusia includens*

**DOI:** 10.1673/031.007.1635

**Published:** 2007-03-29

**Authors:** Dawn M. Olson, Felix L. Wäckers

**Affiliations:** ^1^ Crop Protection and Research Management Unit, USDA-ARS, Tifton, GA 31794, USA, dolson@tifton.usda.gov; ^2^ Netherlands Institute of Ecology, NIOO-CTO, P.O. Box 40, 6666 ZG Heteren, The Netherlands

We investigated whether the age of field margins that had been established for conservation of northern bobwhite populations also enhance biological pest control in adjacent cotton fields. The impact of second year margins on the cotton crop did not significantly differ from first year margins with regard to pests or biological control. Analysis of the gut contents of *M*. *autographae*, suggested that this species is food-limited in the margins, whereas a single vetch species proved to be a good food source. This study shows that non-crop structures designed for a particular ecological function may be unsuitable for other ecological services. By making small adjustments in the vegetative com posit ion, we might be able to effectively stack multiple ecological functions and thereby optimize ecological benefits.

# Research article: Classical biological control in Brazil: *Ageniaspis citricola* x *Phyllocnistis citrella*, a recent case of success

**DOI:** 10.1673/031.007.1636

**Published:** 2007-03-29

**Authors:** J.R.P. Parra, P.T. Yamamoto, M.C. de Chagas, J.M.S. Bento, C. Massari, A.M. Nascimento

**Affiliations:** ^1^ ESALQ/USP - Departamento de Entomologia, Fitopatologia e Zoologia Agrícola, 13418-900, Piracicaba, SP, Brasil, diretor@esalq.usp.br; ^2^ FUNDECITRUS, Araraquara, SP, Brasil; ^3^ EMBRAPA, ENPARNE, Natal, RN, Brasil

The citrus leaf miner *Phyllocnistis citrella* was first referred in our country in 1996. After the introduction of the parasitoid, *Ageniaspis citricola* (Hymenoptera: Encyrtidae) in 1998, it was developed a rearing technique and around 1 million of parasitoids were produced and released mainly in the State of São Paulo, Brazil to control this important pest. Nowadays, 7 years after the parasitoid introduction, *A*. *citncola* is well adapted and it was able to considerably reduce the citrus leafminer infestation, and as a consequence it also contributed for the reduction of citrus canker. The rearing technique will be described, as well as the adaptation of the parasitoid to Brazilian regions with different climatic conditions.

# Research article: Host specificity in the egg parasitoid *Telenomus busseolae* is mediated by sex pheromone compounds

**DOI:** 10.1673/031.007.1637

**Published:** 2007-03-29

**Authors:** Ezio Peri, Salvatore Guarino, Paolo Lo Bue, Alan Cork, Stefano Colazza

**Affiliations:** ^1^ S.En.Fi.Mi.Zo Department, Università di Palermo, Viale delle Scienze 13, 90100 Palermo, Italy, e.peri@unipa.it; ^2^ University of Greenwich, Central Avenue, Chatham Maritime Kent, UK

Several studies showed that egg parasitoids are able to detect host sex pheromones produced by adult hosts as a host cue. In this way female wasps are directed toward an area where host mating is in progress and where an oviposition has probably taken place or is soon to occur. *Telenomus busseolae* Gahan (Hymenoptera: Scelionidae) is a solitary egg parasitoid of various noctuids (Lepidoptera, Noctuidae) belonging to the genera *Sesamia*. In field observations, it has been demonstrated that *T*. *busseolae* reacts to the pheromone emitted by females of the corn stalk borer, *Sesamia nonagrioides* (Lefebvre) and the pink stem borer, *Sesamia calamistis* (Hampson). It is known that the sex pheromone produced by calling lepidopteran females are usually a mixture of compounds. In laboratory experiments, it was shown that three components of the synthetic sex pheromone of the Mediterranean stem borer, e.g. (Z)-11–16:Ac (the main component), (Z)-11–16:Ald and 12:Ac, attract wasp females, while a fourth compound, (Z)-11–16:OH, did not. Interestingly, these active compounds are also present in the sex pheromone blends of other host *Sesamia* species, and in the sex pheromone blends of other non-host noctuid species. To evaluate the role played by some components of noctuid sex pheromone in the host specificity of *T*. *busseolae*, we carried a series of laboratory experiments with a Y-tube olfactometer coupled a video tracking and motion analysis system. In particular we analyzed the behavioural response of *T*. *busseolae* females to the main compounds of the synthetic sex pheromones of the corn earworm, *Helicoverpa armigera* (Hübner). Bioassays were carried out with the whole corn earworm sex pheromone blend [(Z)-9–16:Ald : (Z)-11–16:Ald at ratio of 3 : 97], and the following single compounds: (Z)-9–16:Ald present only in the corn earworm sex pheromone, (Z)-11–16:Ac the main component of the Mediterranean stem borer sex pheromone, and (Z)-11–16:Ald common to both noctuids. All the treatments were bioassayed at two different doses (0.1 mg and 1.0 mg). The results showed that *T*. *busseolae* females werel) attracted by (Z)-11–16:Ac at 0.1 mg and by (Z)-11–16:Ald at 1.0 mg; 2) not attracted by the whole sex pheromone of the corn earn borer at both concentrations; and 3) slightly deterred by (Z)-9–16:Ald at both concentrations. These results suggest that (Z)-9–16:Ald is a key compound which prevents *T*. *busseolae* females responding to the corn earworm sex pheromone.

# Research article: Importation of *Peristenus* spp. for the biological control of *Lygus hesperus* in California

**DOI:** 10.1673/031.007.1638

**Published:** 2007-03-29

**Authors:** Charles H. Pickett, Dominque Coutinot, Kim A. Hoelmer, U. Kuhlmann, H. Goulet

**Affiliations:** ^1^ Biological Control Program, California Department of Food & Agriculture, 3288 Meadowview Rd, Sacramento, CA 95832 USA, cpickett@cdfa.ca.gov; ^2^ USDA, ARS European Biological Control Laboratory, Campus International de Baillarguet, CS 90013 Montferrier-sur-Lez, 34988 St. Gely du Fesc Cedex, France; ^3^ USDA ARS BIIR, 501 S. Chapel St., Newark, DE 19713 USA; ^4^ CABI Bioscience, Rue des Grillons, 2800 Delemont, Switzerland; ^5^ Agriculture and Agri-Food Canada, K.W., Neatby Biding, 960 Carling Ave., Ottawa, ON K1A 06, Canada

*Peristenus digoneutis* during the 1980′s was successfully imported and colonized in alfalfa in eastern United States for the control of *Lygus lineolaris*. Although a similar effort was made in the 1970′s in central California, *Peristenus* spp. failed to establish permanent populations. The reported impact of *P*. *digoneutis* on the east coast, and renewed efforts to import *Peristenus* spp. by Canadians, triggered a renewed and much greater effort to permanently colonize populations of *Peristenus* collected in Europe for control of *L*. *hesperus*, a serious pest of both strawberries and cotton, as well as numerous seed crops in California. Beginning in 1998, *P*. *relictus* (formerally *P*. *stygicus*) and *P*. *digoneutis* were collected from several locations in southern Europe. Both species were released at up to six locations over a 6 year period in central California. More recently, releases have begun in the central coast strawberry growing region. At the original release site in Sacramento, a 0.25 ha plot of alfalfa, both *P*. *digoneutis* and *P*. *relictus* increased in numbers through 2004, three years after the last releases were made, reaching a high of 90%, in late summer. Parasitoids have been recovered from vacant fields of weedy annuals within 2 km of this site. *Peristenus relictus* have been recovered one year after last released into wild vegetation bordering commercial strawberries grown near Castroville on the central coast of California.

# Research article: Towards a thousand sequence ichneumonid wasp molecular phylogeny (Insecta: Hymenoptera: Ichneumonidae) based on the 28S rDNA D2–D3 region

**DOI:** 10.1673/031.007.1639

**Published:** 2007-03-29

**Authors:** Donald L. J. Quicke, Nina M. Laurenne, Mike G. Fitton, Gavin R. Broad

**Affiliations:** ^1^ Department of Entomology, Natural History Museum, Cromwell Road, London SW75BD, and Division of Biology, and Centre for Population Biology, Imperial College London, Silwood Park Campus, Ascot, Berkshire SL5 7PY, UK.; ^2^ Finnish Museum of Natural History, Zoological Museum, Entomological Division, P.O. Box 17(P. Rautatiekatu 13), FIN-00014 University of Helsinki, Finland; ^3^ Department of Entomology, Natural History Museum, Cromwell Road, London SW75BD, UK; ^4^ Centre for Ecology and Hydrology, Monks Wood, Abbots Ripton, Huntingdon PE28 2LS, UK

There is an inevitable trade-off in molecular phylogenetics between the number of genes sequenced and the number of taxa sampled. While having multiple gene fragments to analyse for all taxa would be ideal, the practicality is that seldom do universal primers work on all taxa and for rare species that may only be available from old preserved specimens, DNA degradation means that only multi-copy genes are generally amplifiable. Because of these constraints there has been considerable debate and experimentation over whether it is better to add characters or taxa to a matrix in terms of the accuracy of the final phylogenetic reconstructions, and as far as there is a consensus view, adding taxa, especially ones that help break long branches, appears to have the greater beneficial effect. Our effort has been focused on obtaining a robust molecular phylogeny for the huge parasitic wasp family Ichneumonidae, which has more than 23,000 described species but with an actual species richness ventured to be well in excess of the 60,000 estimated by Townes. Given its size we could not attempt full coverage, but we have tried to get samples from as many different and informative lineages as possible and to sequence these for a single gene fragment initially, the D2–D3 variable region of the nuclear 28S rDNA gene which has been shown to contain a great deal of phylogenetic information within the Ichneumonoidea, despite issues with character alignment (homology). The Ichneumonidae is almost infamous for its taxonomic difficulty and high levels of morphological homoplasy, and while with experience it is fairly easy to assign most specimens correctly to subfamily, DNA is revealing instances where the understanding of subfamily membership based purely on morphology can be extremely misleading. For example, a genus *Zurquilla* Gauld was originally described in the Tryphoninae but it was subsequently shown to be a synonym of the cryptine *Nipponaetes* Uchida, and DNA indeed confirmed that these are in fact members of the Cryptinae. Other similar examples have come light as a result of this study and will be reported elsewhere. Considerable effort has gone in to obtaining specimens for sequencing over a period of more than 10 years, and thanks to the help of many collaborators we have amassed material of all subfamilies, all taxa that have ever been afforded subfamily level status, virtually all tribes, and most of the taxa whose taxonomic positions have been questioned. Thus of the recognised tribes (http://iris.biosci.ohio-state.edu/catalogs/ichneumonids/master_list2.html) our data set is currently lacking only Ankylophonini (Tryphoninae), known from a single species from one locality in Australia; Xenothyrini (Labeninae), based on a single species from Brazil; Clypeodromini (Ichneumoninae), based on a single Palaearctic genus; Eurylabini (Ichneumoninae), based on a single Palaearctic and Oriental genus; and the Goedartiini (Ichneumoninae), based on three small Palaearctic and Oriental genera. Monophyly of the Ichneumoninae (excluding the Alomyinae; Laurenne NM. et al. In press. *Cladistic*) is strongly supported and therefore the absence of three of its constituent tribes from our data set will hopefully have little effect on the overall picture of ichneumonid relationships recovered. In order to be able to include some taxa for which only old dry specimens were available, we have employed a set of internal primer pairs that each amplify just a short fragment of the gene. This has enabled us to include some very rare taxa whose morphology is ambiguous in terms of their systematic placement (Townes H K. 1969. *Memoirs of the American Entomological Institute* 11, 1–300), e.g. *Lapton* Nees, *Ischyrocnemis* Holmgren, *Apolophus* Townes and *Bremiella* Dalla Torre, together with other rare entities such as the ctenopelmatines *Hypopheltes* Cushman and *Westwoodia* Brullé, and the acaenitines *Yamatarotes* Uchida and *Jezarotes* Uchida. Primers used (5′ – 3′) and approximate amplicon sizes were as follow: D2-only (Fwd.: AGAGAGAGTTCAAGAGTACGTG, rev.: TTGGTCCGTGTTTCAAGACGGG - 490 bps), D2-D3A (Fwd.: GCGAACAAGTACCGTGAGGG, rev.: TAGTTCACCATCTTTCGGGTC - 730 bps), D2-D3B (Fwd.: AGAGAGAGAGTTCAAGAGTACGTG, rev.: CGCTACGGACCTCCATCA - 730 bps), RJF_int1 (Fwd.: AGAGAGAGAGTTCAAGAGTACGTG, rev.: CGGGTCGCGACGTCCTACTA - 200 bps), RJF_int2 (Fwd.: TAGTAGGACGTCGCGACCCG, rev.: ATACCGTGCGRRTACCGCC - 150 bps), RJF_int3 (Fwd.: GGCGGTAYYCGCACGGTAT, rev.: CCGTGTTTCAAGACGGGT - 230 bps), JM_int1 (Fwd.: AGAGAGAGAGTTCAAGAGTACGTG, rev.: GGTCAGACAACCGRGGGTCTG - 250 bps), JM_int2 (Fwd.: CAGACCCYCGGTTGTCTGACC, rev.: CCAACAGCYGRCCAGGCCCG - 150 bps), and JM_int3 (Fwd.: CGGGCCTGGYCRGCTGTTGG, rev.: GGTCCGTGTTTCAAGACGGGTCCTG - 120 bps). Our current data set comprises over 1000 sequences representing approximately 600 named genera. Some groups such as the Pimplinae show rather little sequence variation, and in analyses this group shows little phylogenetic structure. In contrast, great sequence diversity has been discovered within some genera, notably *Mesochorus* Gravenhorst (Mesochorinae) and *Orthocentrus* Gravenhorst and other genera of Orthocentrinae. The data set is being analysed using a wide range of gap opening and gap extension parameters in Clustal X and the resulting alignments analysed using the programme TNT. Consistent features of analysis of both manually and Clustal X-aligned data sets are as follows. Basal positions for the Xoridinae and Labeninae and also often the Lycorininae, though in the latter case this might be due to long branch attraction. Then a split into two major clades, one comprising the Pimpliformes (sensu Gauld ID [1997. *Memoirs of the American Entomological Institute* 57, 1–485] but also including Collyriinae) together with the Ichneumoniformes sensu Quicke et al. (Quicke DLJ et al. 2000. In A. D. Austin & M. Dowton, editors, *Hymenoptera*: *Evolution*, *Biodiversity and Biological Control.* Pp 74–83. Canaberra: CSIRO) (i.e. Ichneumoninae + Cryptinae + Alomyinae + Adelognathinae), plus Eucerotinae, Claseinae, Pedunculinae, Brachycyrtinae and Agriotypinae, with the first four of these subfamilies often forming a ‘Gondwanan group’ (see Laurenne et al., in press). In addition the Microleptinae sensu stricto were often recovered basal to the Ichneumoniformes. The other major clade comprises the Ophioniformes group sensu Quicke et al. (Quicke DLJ et al. 2005. *Journal of Natural History* 39, 2559–2578; Quicke DLJ et al. 2000. In A. D. Austin & M. Dowton, editors, *Hymenoptera*: *Evolution*, *Biodiversity and Biological Control.* Pp 74–83. Canaberra: CSIRO) - i.e. including Ophioninae, Ctenopelmatinae, Banchinae, Mesochorinae, Nesomesochorinae, Nonninae, Metopiinae, Campopleginae, Tatogastrinae, Cremastinae, Tersilochinae, Anomaloninae, Neorhacodinae, Oxytorinae, Phrudinae and Stilbopinae. In agreement with Quicke et al. (2000. In A. D. Austin & M. Dowton, editors, *Hymenoptera*: *Evolution*, *Biodiversity and Biological Control.* Pp 74–83. Canaberra: CSIRO), Orthopelmatinae was consistently placed between the two major clades. Several putatively monophyletic subfamilies were only recovered as monophyletic under restricted ranges of parameter values or not at all. These include the Banchinae (the *Banchus* Fabricius genus group and *Townesion* Kasparyan often being placed separately), Ctenopelmatinae (Scolobates Gravenhorst and *Physotarsus* Townes [Scolobatini sensu stricto - i.e Westwoodiini excluded] often excluded), Tryphoninae (*Ibornia* Seyrig never recovered with rest, and sometimes *Idiogramma* Förster recovered separately), Metopiinae (*Colpotrochia* Holmgren often recovered separately). However, that each of these taxa are recovered as monophyletic in some analyses is seen as strong support that they are indeed natural groups. In the case of *Colpotrochia* we suspect that this is due to long branch effects; in the case of *Ibornia* we are less certain. The Phrudinae were never recovered as monophyletic and while *Phrudus* Förster and close relatives were often recovered with or close to the *‘Melanodolius’* Saussure group, *Brachyscleroma* and *Seleucus* Holmgren appear to be quite unrelated. The bulk of Phrudinae were often recovered in the same clade as the Tersilochinae, and these were placed rather basally in the Ophioniformes. Oxytorinae were usually recovered within the Ctenopelmatinae, but of the latter, the Scolobatini sensu stricto were usually excluded and instead placed near the base of the Cremastinae. We hope the resulting molecular phylogenies will stimulate further work on this family including combined molecular and morphological studies. It provides a clear, if not fully resolved, framework for future work, and importantly highlights groups for which acquisition of additional data (mainly additional genes but also various critical taxa) are a priority.

# Research article: A survey in Africa for potential biocontrol agents of the *Erythrina* gall wasp, *Quadrastichus erythrinae*, a new invasive species in the Pacific Region

**DOI:** 10.1673/031.007.1640

**Published:** 2007-03-29

**Authors:** Mohsen M. Ramadan

**Affiliations:** State of Hawaii Department of Agriculture, Division of Plant Industry, USA, mohsen.m.ramadan@hawaii.gov

The erythrina gall wasp (EGW), *Quadrastichus erythrinae* Kim (Hymenoptera: Eulophidae), invaded Hawaii in April 2005 and has rapidly dispersed throughout all the major Hawaiian Islands in less than a year. Severe infestations have devastated native and introduced trees of the genus *Erythrina* (Family Fabaceae) in the Western Pacific and Hawaii (Hawaii, Guam, Taiwan, Singapore, Thailand, Philippines, Japan, and China). Pruning and chemical control measures have failed to contain this pest and biocontrol is thought to be the only long-term solution. An exploratory trip in Tropical East Africa was undertaken during December 05 and January - February 06 to determine the origin of this pest and survey for its associated parasitoids in its native range. Tanzania was selected as the starting point of exploration because of the number of *Erythrina* species logged to be endemic to this country; more than anywhere else in Africa. Leaves and stems of nine *Erythrina* species were lightly attacked by gall wasps. Examination of the gall formers of *E*. *abyssinica*, and *E*. *latissima* from certain localities showed its great similarity to the gall former of *E*. *variegata* in Hawaii. The female wasps differ only in the coloration of the hind legs; however, formal determination is still pending. *Erythrina* trees in Tanzania were very healthy and showed few leaves with galls. A handful of hymenopteran parasitoids, three of which are the major biotic mortality factors in Tanzania, parasitized most of the wasp immature stages in the galls. They are ectoparasitoids; a eurytomid (*Eurytoma* sp.) and two eulophids (*Aprostocetus* spp.), whose larvae develop within the galls on larvae and pupae of the gall former. Seven shipments of infested *Erythrina* leaf and stem samples were shipped to the Hawaii Department of Agriculture Insect Quarantine Facility for parasitoid emergence. Parasitoids from the Tanzanian collection and a similar *Eurytoma* sp. from Kwazulu-Natal, South Africa, were amenable for insectary rearing on EGW from Hawaii. Experiments are being conducted to understand the biology of these parasitoids and determine its host range. Host specificity testing will involve determination of the response of these parasitoids to beneficial gall formers already established in Hawaii for weed biocontrol, native gall formers, and representatives of other insects residing in plant tissues.

# Research article: Potential of *Aganaspis daci* (Hymenoptera: Eucoilidae) for biological control of tephritid fruit flies

**DOI:** 10.1673/031.007.1641

**Published:** 2007-03-29

**Authors:** Mohsen Ramadan, Aime Bokonon-Ganta, Roger Vargas, Nikos Papadopoulos

**Affiliations:** ^1^ State of Hawaii Department of Agriculture, Division of Plant Industry, Plant Pest Control Branch, 1428 South King Street, Honolulu, Hawaii 96814 (USA), mohsen.m.ramadan@hawaii.gov; ^2^ University of Hawaii at Manoa, College of Tropical Agriculture and Human Resources, Department of Plant and Environment Protection Sciences, 3050 Maile Way, Honolulu, Hawaii 96822(USA).; ^3^ USDA-ARS, US Pacific Basin Agricultural Research Center, P.O. Box 4459, Hilo, HI 96720.; ^4^ Department of Agriculture Crop Production and Rural Environment, Laboratory of Entomology and Applied Zoology, Phytokou St. 384 46 N. lonia Magnisias, Greece.

Host range studies and methods for rearing the larval-pupal endoparasitoid, *Aganaspis daci* (Weld) were investigated under quarantine conditions in Hawaii. The parasitoid developed successfully in *Ceratitis capitata*, *Bactrocera dorsalis*, *B*. *latifrons*, and *B*. *cucurbitae* (Diptera: Tephritidae). Parasitism of the late instar *B*. *cucurbitae* produced less parasitoid progeny than other tephritid hosts, and *B*. *latifrons* was the optimum host for insectary rearing. Studies on non-target effects of *A*. *daci* showed that female wasp had negative responses to the flower-head feeders and gall forming tephritids in Hawaii. Results of immature development, reproductive potential, and optimum rearing protocols will be discussed.

# Research article: Status of the Japanese beetle parasitoid *Tiphia vernalis* Rohwer in Connecticut

**DOI:** 10.1673/031.007.1642

**Published:** 2007-03-29

**Authors:** Darryl Ramoutar, Ana Legrand

**Affiliations:** Department of Plant Science, U-4067, 1376 Storrs Road, University of Connecticut, Storrs, CT 06269 USA, ana.legrand@uconn.edu

During 1920′s and early 1930′s USDA entomologists imported *Tiphia vernalis* Rohwer (Hymenoptera Tiphiidae) from Korea for Japanese beetle control. Numerous wasp releases were made throughout the Northeast. In six of Connecticut's eight counties, releases of *T*. *vernalis* were made between 1936 and 1949. Since 1950 the occurrence of *T*. *vernalis* in Connecticut had not been monitored and some literature had suggested that these wasps were rare in the state. This study was undertaken to determine the distribution and seasonal occurrence of *T*. *vernalis* in Connecticut. In 2004, at least one golf course in each county was surveyed in Spring. In 2005, golf courses in the north, central and south portions of the state were surveyed for six weeks to determine the seasonal occurrence of *T*. *vernalis* adults. *T*. *vernalis* wasps were found in every county of the state. The 2005 survey results indicate that *T*.*vernalis* adults are active from the first week in May to the beginning of June. The peak numbers were observed in the fourth week of May. There was substantial variation in the numbers of wasps observed at each site. For example, in Hunter Memorial Park (New Haven Co.) the peak number observed was 563 adults while only 28 were seen in Skunkgamaug Golf Course (Tolland Co). The results of this survey show that the wasps are not rare in Connecticut and that they should be integrated with other measures for Japanese beetle management. For example, the public is generally unaware of these wasps and IPM guidelines could suggest methods to conserve and enhance their populations. While adult wasps were readily observed in this study, parasite larvae were not found in the limited search for parasitized Japanese beetle larvae. Additional work needs to address the current rates of parasitism by *T*. *vernalis*.

# Research article: Why do mostly-predaceous bugs sometimes plant feed and does it matter?

**DOI:** 10.1673/031.007.1643

**Published:** 2007-03-29

**Authors:** Bernard Roitberg, David Gillespie, Robert McGregor

**Affiliations:** ^1^ Department of Biological sciences, Simon Fraser University, Burnaby, BC, V5A 1S6 Canada, roitberg@sfu.ca; ^2^ Pacific Agri-Food Research Centre, Agriculture and Agri-Food Canada, POB 1000 Agassiz, BC VOM 1A0 Canada; ^3^ Douglas College, New Westminster, BC V3L 5B2 Canada

Why do omnivorous mirids feed on plants? There are at least 3 reasons: (i) Plant food can be an alternative to animal food “Equivalence”, (ii) plants can provide a critical resource required for predation “Facilitation”, or (iii) plants can provide essential nutrients not available in animal prey “Independence”. We conducted a series of factorial experiments where we deprived individual *Dicyphus hesperus* bugs of various combinations of plant and prey and then measured their response to plant (of varying quality) and prey resources. If omnivores do feed on plants for specific reasons as suggested above does that matter in the big scheme of things i.e. predator-prey dynamics? We developed an analytical model that incorporates the aformentioned different plant feeding mechanisms by the omnivore in a plant-herbivore-omnivore system. The results clearly show that raison d'etre for plant feeding can have substantial impact on prey (pest) dynamics. Given that omnivory is very common in the real world, we close with a discussion of plant feeding by omnivores as biological control agents.

# Research article: Changes in the biochemistry of the host hemolymph *Diatraea saccharalis* (Lep.: Crambidae) induced by the endoparasitoid *Cotesia flavipes* (Hym.: Braconidae)

**DOI:** 10.1673/031.007.1644

**Published:** 2007-03-29

**Authors:** G. Salvador, F.L. Cônsoli

**Affiliations:** Department of Entomology, ESALQ, University of São Paulo, 13418-900, Piracicaba, SP, Brasil, fconsoli@esalq.usp.br

Koinobiont parasitoids induce changes in the composition of the host internal environment to adequate the nutrients available to the specific requirements of the developing immature. *Cotesia flavipes* is an important biological control agent employed in applied programs for the control of the sugarcane borer in extensive areas in Brazil. They are mass produced on the natural host, and the increase in demand of renewable sources of energy will require the development of cost effective rearing technologies. Such technologies will certainly require information on the nutritional needs of such species, and the changes parasitoids induce on their host are a reliable source of information to understand the nutritional ecology of these insects. Therefore, we have evaluated changes of the major macromolecules in the hemolymph composition of the host throughout parasitoid development. Host proteins availability is altered early in parasitoid development, while changes in sugar and lipid levels will occur only five days after parasitization. All nutrients were found in much lower concentration in the hemolymph of parasitized than from non-parasitized hosts. We discuss the importance of such changes in supporting parasitoid development.

# Research article: Cell-free immune reactions in insects

**DOI:** 10.1673/031.007.1645

**Published:** 2007-03-29

**Authors:** Otto Schmidt, Gang Ma, Harry L. S. Roberts, M. Mahbubur Rahman

**Affiliations:** Insect Molecular Biology, University of Adelaide, Glen Osmond, SA 5064, Australia, otto.schmidt@adelaide.edu.au

Insects, like many other multicellular organisms, are able to recognise and inactivate potential pathogens and toxins in the absence of cells. We have reported that the recognition and inactivation of lipopolysaccharides (LPS) and bacteria is mediated by lipophorin particles, which are the lipid carrier in insects. In immune-induced insects sub-populations of lipophorin particles are associated with pattern recognition proteins and regulatory proteins that activate prophenoloxidase. Moreover, interactions with lectins result in the assembly of lipophorin particles into cage-like coagulation products, effectively protecting the surrounding tissues and cells from the potentially damaging effects of pathogens and phenoloxidase products. The existence of cell-free defence reactions implies that immune signals exist upstream of cell-bound receptors.

# Research article: Are insect immune suppressors driving cellular uptake reactions?

**DOI:** 10.1673/031.007.1646

**Published:** 2007-03-29

**Authors:** Otto Schmidt, Harry L.S. Roberts, Richard V. Glatz, Sassan Asgari

**Affiliations:** ^1^ Insect Molecular Biology, School of Agriculture and Wine, University of Adelaide, Glen Osmond, SA 5064, Australia, otto.schmidt@adelaide.edu.au; ^2^ CSIRO HSN, Adelaide, SA 5000, Australia; ^3^ Department of Zoology and Entomology, School of Life Sciences, University of Queensland, St Lucia QLD 4072, Australia

Many insect parasitoids that deposit their eggs inside immature stages of other insect species inactivate the cellular host defence to protect the growing embryo from encapsulation. Suppression of encapsulation by polydnavirus-encoded immune-suppressors correlates with specific alterations in hemocytes, mainly cytoskeletal rearrangements and actin-cytoskeleton breakdown. We have previously shown that the *Cotesia rubecula* polydnavirus gene product CrV1 causes immune suppression when injected into the host hemocoel. CrV1 is taken up by hemocytes although no receptors have been found to bind the protein. Instead CrV1 uptake depends on dimer formation, which is required for interacting with lipophorin, suggesting a CrV1-lipophorin complex that is internalised by hemocytes. Since treatment of hemocytes with oligomeric lectins and cytochalasin D can mimic the effects of CrV1, we propose that some dimeric and oligomeric adhesion molecules are able to cross-link receptors on the cell!

# Research article: Development of a rearing technique for the parasitoid *Tamarixia radiata* (Hym., Eulophidae) to control the psyllid *Diaphorina citri*

**DOI:** 10.1673/031.007.1647

**Published:** 2007-03-29

**Authors:** M.L.G. Torres, J.R.P. Parra, D.E. Nava

**Affiliations:** ESALQ/USP Departamento de Entomologia, Fitopatologia e Zoologia Agrícola, 13418-900, Piracicaba, SP, Brasil, diretor@esalq.usp.br

The greening disease was first detected in Brazil in 2004, and this work was carried out to develop a parasitoid rearing technique for releasing in the areas where the disease is a serious problem in the State of São Paulo. The rearing technique for *Diaphorina citri* was adapted from that developed for the citrus leafminer for parasitoid production. *Tamarixia radiata*, the parasitoid of the psyllid, was first detected in Brazil only recently, but surveys have indicated it is widespread though the citrus orchard areas, including those were the greening disease is occurring. The parasitization is very high in some areas of the State, and we have initiated our release program to evaluate how efficient this natural enemy can be in reducing the psyllid population, and consequently, the spread of the disease through the citrus growing areas.

# Research article: Tailor-made learning and memory in parasitoids

**DOI:** 10.1673/031.007.1648

**Published:** 2007-03-29

**Authors:** Louise E.M. Vet, Hans M. Smid

**Affiliations:** ^1^ Netherlands Institute of Ecology (NIOO-KNAW), P.O.Box 1299, 3600 BG Maarssen The Netherlands, l.vet@nioo.knaw.nl; ^2^ laboratory of Entomology, Wageningen University, Binnenhaven 7, 6709 PD Wageningen, The Netherlands

Learning in insect parasitoids is a research field that has grown considerably over the last decade, with emphasis on chemical ecology and behaviour. Studies have commonly addressed the effect of experience on the response to specific stimuli, and have yielded hypotheses on the adaptive value of such learning in foraging parasitoids. Only recently have researchers begun to use parasitoids to study the mechanistic aspects of learning, becoming aware that to fully understand variation in learning an interdisciplinary approach is needed whereby mechanistic, ecological and evolutionary studies are combined. We believe parasitoids to be excellent model systems to study the evolution of information processing and cognition. Their diversity in ecological function combined with a relatively narrow-tuned behavioural task is expected to result in prominent adaptations in learning and memory. Differences in the expression of learning between closely related species create excellent opportunities to study species-typical learning. We find such an opportunity in our model system of two co-existing *Cotesia* parasitoid species that occupy slightly different niches. In the Netherlands, *Cotesia glomerata* mainly attacks the gregariously feeding caterpillars of the large cabbage white *Pieris brassicae* while *Cotesia rubecula* is specialized on the solitarily feeding small cabbage white *Pieris rapae*. After an oviposition experience in a caterpillar on a previously unattractive host plant, *C*. *glomerata* shows a preference for the odours of that plant. In *C*. *rubecula* we could not detect such a shift in plant odour preference after an ovi posit ion experience. We first investigated if this difference in learning was due to differences in their olfactory pathways, i.e. the sensory perception of plant odours, their antennal olfactory sensilla or anatomy of the antennal lobe, but could not find a clear indication. We then focussed on the learning mechanism and memory formation and show here, for the first time, that there is a clear interspecific difference in the functional requirements for the formation of long-term memory (LTM). *C*. *glomerata* learns fast and already forms LTM after a single learning event, while *C*. *rubecula* is a slow learner that needs three spaced learning events to change its plant preference. We argue that this inter-specific variation in LTM acquisition reflects a difference in the wasp's searching behaviour in nature, related to the distribution of their caterpillar hosts. We expect such tailor-made learning and memory to be more common in parasitic wasps than presently assumed.

# Research article: Using HPLC Sugar Analysis to Study Nectar and Honeydew Feeding in the Field

**DOI:** 10.1673/031.007.1649

**Published:** 2007-03-29

**Authors:** Felix Wäckers

**Affiliations:** Centre for Sustainable Agriculture, Lancaster Environment Centre Lancaster Univ., Lancaster LA14YQ, UK, f.wackers@lancaster.ac.uk

Many entomophagous arthropods depend on carbohydrate-rich food sources such as nectar or honeydew as a source of energy. Nevertheless, we often know little about the extent to which various sugar sources contribute to the diet of predators and parasitoids. One way to study food use in the field is to analyze guts of collected insects for source-specific compounds. Sugar sources often show distinct differences in their carbohydrate composition. Especially honeydew often features a broad range of phloem-feeder synthesized di-and oligosaccharides. HPLC sugar analysis can be used to study sugar sugar levels in individual field collected insects. I will explore how we can interpret information from these analyses to characterize the nutritional state and feeding history of a range of entomophagous insects.

# Research article: Host plant infection by a plant pathogen changes parasitoid host-searching behavior

**DOI:** 10.1673/031.007.1650

**Published:** 2007-03-29

**Authors:** Liande Wang, Stefan Vidai

**Affiliations:** ^1^ Institute of Applied Ecology & Key Laboratory of Biopesticide and Chemical Biology, MOE. Fujian Agriculture & Forestry University, Fuzhou, 350002, P.R. China, wangliande@yahoo.com; ^2^ Department of Crop Science, Entomological Section, Georg-August-University, Goettingen, 37077, Germany

Plants frequently suffer from either simultaneously or sequentially infestations or attack by plant pathogens and/or insect herbivores. When damaged, plants release volatile compounds (VCs), which are used by parasitoids to locate their herbivorous hosts. However, few studies so far investigated the influence of simultaneous colonisation of plants by plant pathogens and insect herbivores on the efficacy of parasitoids to locate their hosts. We used a series of laboratory and greenhouse experiments using tomato plants, the powdery mildew *Oidium neolycopersici*, whiteflies (*Trialeurodes vaporariorum*) and the parasitoid *Encarsia formosa*, respectively, to test the impact of diseased host plants on herbivore-parasitoid-interactions. *Encarsia* females were significantly less attracted to powdery mildew infected tomato plants (PMP) as compared to healthy plants (HP); thus parasitism rates of whiteflies (WF) were significantly lower on PM as compared to HP (17.5:41.5 %). These differences disappeared when *E*. *formosa* females were forced to parasitize whiteflies on either PMP or HP. The performance of the parasitoid was not significantly influenced when developing on whiteflies feeding on powdery mildew infected plants. In olfactometer bioassays, either naïve or experienced parasitoids significantly preferred healthy plants colonised by whiteflies (HPWF) as compared to powdery mildew infected plants colonised by whitefly (PMPWF) (30.0:10.0% for naïve parasitoids; 41.4:15.2% for experienced parasitoids). In wind tunnel bioassays volatiles emanating from WFs significantly increased upwind oriented flight and landing in *Encarsia* targets as compared to any other treatments. Volatiles from HP, PMP, PMPWF and WF, respectively were collected and analyzed by GC and GC-MS, revealing that both qualities and quantities of volatile compounds differed to a large extend. Plants significantly increased volatile emission after being attacked by whiteflies (275.3 mol.hr -1) or inoculated by powdery mildew (267.58 mol.hr-1). However, plants significantly reduced volatile emissions in PMWF co-existing systems (80.58 mol.hr -1).Our results demonstrate that volatiles released by either herbivore damaged plants, pathogen diseased plants or combinations of these agents, respectively, may influence the foraging behavior of the parasitoids while searching for their hosts. The negative impact of diseased host plants on the parasitoids host-searching behavior resulted in reduced efficacies of parasitoids. This in turn may impact the outcome of biological control systems, especially in greenhouse conditions.

# Research article: The wooden anchor

**DOI:** 10.1673/031.007.1651

**Published:** 2007-03-29

**Authors:** Bob Wharton

**Affiliations:** Department of Entomology, Texas A&M University, College Station, TX 77843 USA,rawbaw2@tamu.edu

Despite the enormous diversity of morphological and biological features within the Apocrita, there is widespread support for the monophyly of this group. This incredible diversity is suggestive of a rapid or explosive radiation which, unfortunately, has posed challenges for understanding relationships among the major clades that com prise the Apocrita. Inferences about biological transitions within the Apocrita nonetheless continue to be made by overlaying traits such as ectoparasitism and endoparasitism on trees portraying apocritan relationships. The utility of exploring biological transitions using terms such as koinobiont, idiobiont, ectoparasitoid, and endoparasitoid is examined in light of what we do know about apocritan relationships. The choice of host stage attacked is an important consideration since several putatively basal taxa oviposit into the eggs of their host. A related consideration is the transition from a concealed, wood-inhabiting host to concealed and exposed hosts occupying a diverse array of habitats. Both involve a consideration of the equipment used in host location.

# Research article: Why should a polyembryonic wasp self-superparasitize?

**DOI:** 10.1673/031.007.1652

**Published:** 2007-03-29

**Authors:** Jennifer A. White, D.A. Andow

**Affiliations:** ^1^ Department of Entomology, University of Arizona, 410 Forbes Bldg, Tucson, AZ 85721 USA, jenwhite@email.arizona.edu; ^2^ University of Minnesota, 219 Hodson Hall, 1980 Folwell Avenue, St. Paul, MN 55108 USA

Polyembryonic wasps have eggs that divide asexually within the host to produce broods of clonally identical offspring. For such a species, there is apparently little reason to lay multiple eggs within a host, yet we have found evidence that the polyembryonic species *Macrocentrus grandii* almost always invests additional time and, if possible, eggs into hosts that it has already stung. We investigated and found support for three potential benefits that can be gained by multiple stings. First, multiple stinging allows production of mixed sex broods. We found that 29% of multiply-stung versus 0% of singly-stung hosts produced mixed sex broods. Second, multiple stings decrease the chance of complete brood failure. Singly-stung hosts were 3X more likely to fail to produce broods (through host pupation or death) than multiply-stung hosts. Third, multiple stings increase brood size, within limitations: a second sting resulted in a 50% increase in adult progeny produced per brood, but third and subsequent stings resulted in no increase in adult progeny relative to broods from singly-stung hosts. These results therefore indicate substantial benefit for a second sting, but little benefit for additional stings, particularly when progeny/egg ratios are calculated. However, we also have results that indicate that within-host larval competition is prevalent, suggesting that supernumerary stings may pay off in scramble competition against conspecific larvae. Such additional investment within a single host would be particularly beneficial when hosts, rather than eggs, are limiting.

# Research article: Geometric morphometric characterization of morphological differentiation among cryptic species in the *Aphelinus varipes* complex

**DOI:** 10.1673/031.007.1653

**Published:** 2007-03-29

**Authors:** James, B. Woolley, Angela M. I. de Farias, Jung-Wook Kim, Keith R. Hopper, John M. Heraty

**Affiliations:** ^1^ Department of Entomology, Texas A&M University, College Station, TX, jimwoolley@tamu.edu; ^2^ Federal University of Pernambuco, Department of Zoology, Recife, Brazil; ^3^ North Carolina State University, Department of Entomology, Raleigh, NC; ^4^ USDA-ARS-BIIR, 501 S. Chapel Street, Newark, DE; ^5^ Department of Entomology, University of California, Riverside, CA

We used geometric morphometric methods to study differentiation in antennae, fore wings and mesosoma for male and female specimens from populations in the *Aphelinus varipes* complex from various geographical regions and host species, representing at least five cryptic species. We measured landmark locations of antennal segments, forewings, and mesosoma 462 specimens using a digital camera and Optimas image analysis software. Multivariate statistical analysis of distances between landmark loccation using principal component and canonical variates analysis showed that morphological differentiation between these cryptic species was consistent and significant. However, characterization and visualization of these differences is difficult using distance-based methods. Thus, we used geometric morphometrics, which can be used to study variation in size and shape of biological forms by statistical analysis of the locations of landmark points only, without reference to distances calculated between landmarks. MANOVA showed a significant sex effect so females and males were separately analyzed for each body region. Populations differed for all body regions in both males and females. Both sexes showed significant host effects on morphology for all of the body regions except male pedicel. Discriminant analysis performed well for both male and female mesosoma, but poorly for wings and antennal regions. Discriminant analysis based on wing and antennal distances was much more successful. The third funicular segments of males have two distinct shapes and those of females have three distinct shapes. In forewings, the most significant shape differences were in the delta area. Females and males had similar shape changes. Shape differences in the mesosoma were found at the anterior and posterior margins, as well as in the position of anterior and posterior scutellar setae. Females and males had similar shape deformations compared to the consensus form.
